# Comparison of standard laminectomy with an optimized ejection method for the removal of spinal cords from rats and mice

**DOI:** 10.1179/014788813X13756994210382

**Published:** 2013-09

**Authors:** Heather S Kennedy, Charles Jones, Patrick Caplazi

**Affiliations:** Genentech, Inc., 1 DNA Way, MS 462a, South San Francisco, CA, California, USA

**Keywords:** Ejection, Histotechnology, Laminectomy, Mouse, Rat, Rodent, Rodent necropsy, Spinal cord

## Abstract

For researchers seeking to collect spinal cord samples from mice and rats while avoiding acid decalcification, few options are available. Laminectomy is the standard method which yields high quality samples, yet is time consuming and technically difficult. Ejection of the cord from the vertebral column is another technique commonly used; however, the literature suggests that this method can damage the spinal tissues and is typically avoided when histology of samples is the desired endpoint. Here, we describe an optimized method for ejection of spinal cords from rats and mice, and compare histological quality of these samples with those collected via laminectomy. Our results show that ejection can yield high quality spinal cord samples and may be suitable for use as an alternative to laminectomy.

## Introduction

The removal of spinal cord samples from the spines of mice and rats tends to be technically difficult and time consuming. In order to circumvent this problem, it is possible to process spinal cords enclosed in the spinal canal by including a decalcification step. Because some downstream assays are sensitive to acid decalcification,^1^,^2^ removal of the cords from their bony enclosures prior to processing is preferable, or in some instances necessary. Two methods are commonly used for the collection of spinal cord samples free of bone: laminectomy and ejection. Laminectomy performed by a skilled technician yields spinal cord samples of superior quality.^3^ Unfortunately, laminectomy is tedious and not suitable for collection of large numbers of samples. By contrast, ejection allows rapid collection of large sample numbers; however, particular care must be taken not to damage the delicate cord tissue.^4^ Collection of spinal cords by extrusion under pressure has been described for the rat^1^ and is widely used, although detailed technical accounts that consistently yield high-quality samples in rats and mice are not commonly available in the published literature. Here, we describe an optimized and standardized method for spinal cord ejection from rats and mice, and compare sample quality achieved by ejection with that of samples derived from laminectomy. The optimized ejection technique resulted in spinal cord samples of similar overall histological quality when compared to laminectomy. For many applications, the ejection method described here is a suitable alternative to laminectomy, particularly in instances that require rapid collection of large numbers of samples with the entire cord intact.

## Materials and Methods

### Procedure-necropsy

Animals were maintained in accordance with the *Guide for the Care and Use of Laboratory Animals*^5^ and were housed in facilities accredited by the Association for Assessment and Accreditation of Laboratory Animal Care International. All procedures were approved by Genentech’s IACUC. Adult (7–8 week) female mice, or adult (9–10 week) female rats respectively were arbitrarily assigned to laminectomy and ejection groups. Laminectomy groups (rather than historical data or samples) were included in this study to allow for concurrent identical processing and evaluation of samples eliminating confounding factors for analysis. Group sizes were designed to include replicates for qualitative assessment but not for purpose of statistical analysis. For mice, group sizes were *n* = 6 (laminectomy) and *n* = 8 (ejection), for rats group sizes were *n* = 4 and *n* = 7, respectively.

### Mice – Laminectomy

Mice were anesthetized by intraperitoneal (i.p.) administration of sodium pentobarbital (Nembutal, Ovation Pharmaceuticals, Deerfield, IL, USA; 50–70 mg/kg body mass). Under anesthesia, whole-body transcardial perfusion was performed using phosphate-buffered saline (PBS) solution (10 ml; Sigma-Aldrich, St Louis, MO, USA) followed by 10% neutral buffered formalin (10 ml; Thermo-Fisher Scientific, Waltham, MA, USA). Four per cent paraformaldehyde (10 ml; Sigma-Aldrich) may also be used as an alternative to formalin. Perfusion with fixative prior to collection improves the technique by allowing cleaner dissection and by firming up the neural tissue, thus decreasing the likelihood of damage during removal. Abdominal viscera were removed and discarded. Carcasses were placed in a plastic bag and chilled in wet ice for approximately 20 minutes, enhancing initial fixation of the cord. Access to the CNS was then prepared by removal of the dorsal skin and musculature along the spine. After exposure of the spine, fine-tipped offset bone nippers (Fine Science Tools, Foster City, CA, USA) were used to make a small cut in the lower lumbar spine, typically at the level of the pelvic crests. Using the nippers, the dorsal portion of the vertebral column was dissected from the ventral portion proceeding in a caudal-to-cranial direction, revealing the dorsal aspect of the spinal cord. Upon reaching the occiput, the skull cap was removed in a similar fashion. Next, the brain was gently teased from the cranial cavity in a rostrocaudal direction using micro-dissection iris forceps (Roboz Surgical Instrument Co., Gaithersburg, MD, USA) to tease away the attached cranial nerves. During this step, the brain remains attached to the cord, and is used as a weight to help draw the spinal cord from its vertebral bed. To achieve this, the mice were held vertically with the spine at a slight angle to the cord. While gravitation pulled the brain and cord downward and away from the spine, the cord was slowly eased from the spinal canal using micro-dissection iris forceps or microscissors (Miltex Inc., York, PA, USA). As the lower lumbar region was reached, the isolated CNS was disconnected from the spine by transection and gently placed on a flat surface. After removal of the brain, the spinal cord was put in fixative and fixed for 24 hours prior to processing.

### Mice – Ejection

**Figure 1:**
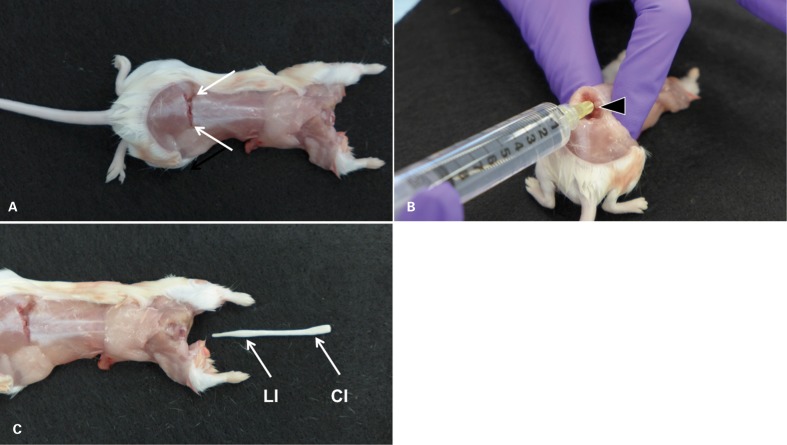
Ejection technique (mouse). (A) The decapitated, partially skinned mouse after application of a transverse cut through the lower lumbar portion of the spine just cranial of the iliac crests (arrows) to expose the lumbar cord. (B) In order to visualize the lumen of the spinal canal, the spine is kinked dorsally by lifting the cranial portion of the mouse firmly held in one hand. With the other hand, a 20 g 1/4″ needle, attached to a 10-ml syringe, is inserted into the spinal canal. (C) Pressure is applied to the plunger of the syringe, resulting in rapid ejection of the entire spinal cord from the cervical opening. Typically, the ejected cord is slightly convoluted. It is shown straightened to demonstrate that it is architecturally intact (absence of tears) and complete, including cervical, thoracic, lumbar, and sacral segments. The entire procedure from applying the lumbar cut to collecting the ejected cord is accomplished in less than 1 minute. CI = cervical intumescence; LI = lumbar intumescence.

Mice were euthanized by carbon dioxide inhalation and immediately exsanguinated by cardiac puncture using a 1-ml syringe with 25 g 1/2″ needle (BD, Franklin Lakes, NJ, USA); exsanguination improves visualization of the insertion point for the cannula used for ejection. Alternatively, whole-body transcardial perfusion may be performed with a PBS solution (10 ml, Sigma-Aldrich, St. Louis, MO, USA). Perfusion with a fixative should be avoided, however, as fixation impedes or prevents ejection of the spinal cord. Using small surgical scissors and forceps (Roboz Surgical Instrument Co.), the dorsal skin was removed. Next, the approximate location of the atlanto-occipital joint was visualized by repeated gentle flexion and extension revealing a shallow groove immediately behind the occiput. Using larger scissors (Roboz Surgical Instrument Co.), animals were decapitated at the alanto-occipital joint, resulting in clear exposure of the cervical spinal cord. If examination is limited to more caudal segments of the spinal cord, precise decapitation is not essential; however, opening of the spinal canal within the cervical region is desirable, since the canal is relatively wide in this portion of the spine and more accessible than at the cervicothoracic junction or within the thoracic segment. After decapitation, a clean transverse cut was made through the lower lumbar portion of the spine, just slightly cranial to the iliac crests (Fig. 1A). The location at the iliac crest is essential for success, as the width of the spinal canal rapidly decreases caudally of this region. Conversely, placing the cut more cranially will result in loss of portions of the lumbar spinal cord, which is often the target of examination (e.g. in experimental allergic encephalitis studies, EAE). Care was taken to avoid fragmentation of the vertebral bone. The transected end of the lumbar cord within the lumen of the spinal canal was visualized by bending the caudal portion of the spine. Excess blood was rinsed from the field with PBS.

A 20 g 1/4″ needle (Air-Tite, Virginia Beach, VA, USA) attached to a 10-ml syringe (BD, Franklin Lakes, NJ, USA) was inserted fully into the lumbar spinal canal (Fig. 1B). The fit should be snug, and a slight twisting motion can be used to work the needle in all the way to the hub. If the needle will not fit, the initial cut may have been placed too far caudally. In this case, a second cut can be placed a few millimeters more cranially. With the needle in place, the carcass was grasped firmly with one hand. Pressure was applied to the syringe plunger with the other hand to release 1–2 ml PBS into the spinal canal, resulting in immediate ejection of the entire cord from the cervical opening. Brain and spinal cord were placed separately in 10% NBF and fixed for 24 hours prior to further trimming and processing.

### Rats – Laminectomy

Rats were anesthetized by i.p. administration of sodium pentobarbital (Nembutal, Ovation Pharmaceuticals; 50–60 mg/kg body mass). Whole-body perfusion was performed using 50 ml of PBS (Sigma-Aldrich) followed by 10% neutral buffered formalin (Thermo-Fisher Scientific; 30–50 ml) or 4% paraformaldehyde (Sigma-Aldrich; 30–50 ml). After removal of the abdominal viscera, carcasses were placed in a plastic bag and chilled in wet ice for approximately 30 minutes. Carcasses were then prepared for removal of the cord as previously described for mice. The vertebrae of rats are much thicker than those of mice, so more force must be applied on the nippers to clip the bone, and extra care taken to avoid slipping and damaging the spinal cord. After reaching the occiput, the skull cap was removed and the brain gently teased from the cranial cavity in a rostrocaudal direction. Removal of the eyes before attempting to pry the brain from the skull may be beneficial, as the intact optic nerves tend to hold the brain in place. For removal of eyes, fine iris forceps (10 cm long with 0.8 mm tip; Roboz Surgical Instrument Co.) are inserted underneath the eye, causing the globe to bulge from the orbit. Forceps are securely braced around the back of the eye. A firm tug will then dislodge the eye from the orbit and sever the optic nerve in the process. Upon exenteration of the brain, the carcasses were held in near vertical position at a narrow angle to the cord. Taking advantage of the gravitational pull on the brain, the entire spinal cord was dissected from the vertebral canal. Brain and spinal cord were placed separately in 10% NBF and fixed for 24 hours prior to further trimming and processing.

### Rats – Ejection

Rats were euthanized by carbon dioxide inhalation and terminal exsanguination was performed by cardiac puncture using a 10-ml syringe with 22 g 1″ needle (BD). As with mice, transcardial perfusion with PBS may be performed. Using large surgical scissors and forceps (Roboz Surgical Instrument Co.), the skin was removed from the entire dorsal portion of the carcass, the location of the atlanto-occipital joint identified by repeated flexion and extension and animals decapitated at the alanto-occipital joint using scissors. Next, the epaxial musculature of the lumbar region was dissected transversally using surgical scissors in order to mark and visualize the area where the injection needle was to be inserted. Using small bone rongeurs (Roboz Surgical Instrument Co.), a clean transverse cut was made through the lower lumbar portion of the spine, just slightly cranial to the iliac crests. As for mice, placement of the transverse cut caudally of the iliac crests may result in insufficient widths of the spinal canal for placement of the ejection needle. Repeated scissoring motions should be avoided in order to prevent fragmentation of the bones and thus difficulty with visualizing the lumen of the spinal column. The caudal portion of the carcass was bent downward and away from the rest of the body. Any additional musculature impeding this step was cut away with scissors. The lumen of the spinal canal should now be apparent, although minor clearing of blood or bone fragments may be necessary in order to reveal it.

Ejection of the cord was achieved by application of 2–8 ml of PBS from a 10-ml syringe (BD) using an 18 g 1/4″ needle (Air-Tite) completely inserted into the lumen of the lumbar spine. Instances where spinal cord remained attached to the carcass by the dura at the cervical opening required minimal additional dissection. Brain and spinal cord were placed separately in 10% NBF and fixed for 24 hours prior to further trimming and processing.

### Histology

Fixed samples were prepared for processing by dividing them into cervical, thoracic, and lumbar sections using single-blade razors (VWR, Radnor, PA, USA). Two or more segments of each region were placed in uni-cassettes (Sakura, Torrance, CA, USA) on biopsy sponges (Mercedes Medical, Sarasota, FL, USA) to preserve orientation during processing. The blocks were sectioned at 4 μm and stained with haematoxylin and eosin. Animal and group identities were blinded prior to evaluation. Evaluation was performed by light microscopy. Three sections per animal were examined representing the cervical, thoracic, and lumbar segments collected. Scores for presence of specific artifacts on a scale of 0 to 3 (Table 1) were assigned for each section. Additional observations were captured in clear-text notes.

**Table 1:** Histology scoring table for sample quality

Score	Quality	Features
0	poor	portions of sample missing or disrupted by tears or clefts; overall architecture distorted at low magnification
1	fair	all architectural features intact; may have several small artifacts (distortion, peripheral portions of tissue lost) that do not impede evaluation
2	good	as 1, but artifacts attributable to collection are rare
3	excellent	no artifacts attributable to collection observed

## Results

### Mice

**Figure 2:**
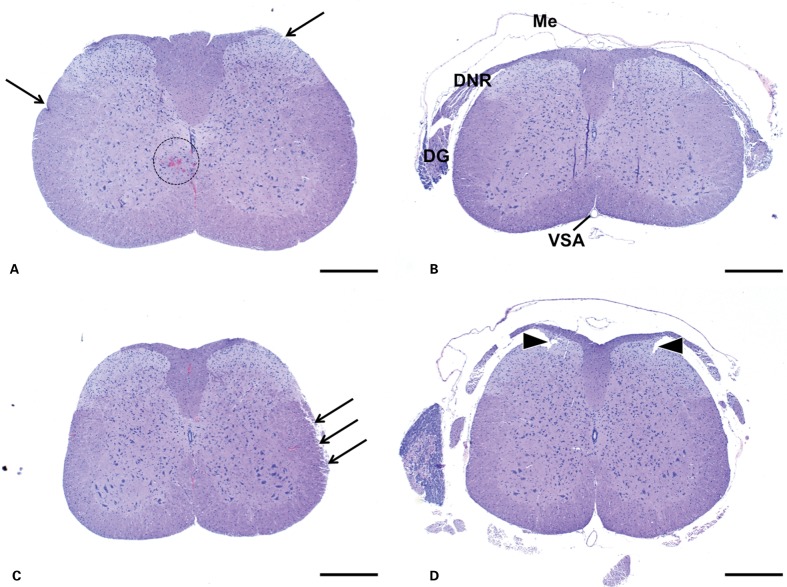
Mouse. Spinal Cord, transverse, H+E. (A), (B) cervical. (C), (D) lumbar. Samples collected via ejection (A), (C) are devoid of meninges and other structures such as spinal nerves. These structures are generally at least in part preserved in samples collected by laminectomy (B), (D). Otherwise preservation of morphological detail is comparable between the methods. Small artifacts that may be present in samples collected by ejection (arrows) include slight superficial fraying, occasional indentations, and small areas of extravasated blood (dashed circle). Artifacts more commonly seen in samples collected by laminectomy include small tears near nerve roots (arrowheads). Me = meninges; DNR = dorsal nerve root; DG = dorsal root ganglion; VSA = ventral spinal artery. Bars = 500 μm.

In mouse spinal cord samples collected via laminectomy, the overall architectural and cytologic detail was very well preserved, consistently achieving quality scores of 2 or 3. Leptomeninges and nerve roots were apparent in most sections and marginal fraying was rarely evident. In mouse spinal cord samples collected via ejection, the overall architectural and cytologic detail was largely preserved; none of the sections had substantial artifacts (score 0). On rare occasions, the neuropil was disrupted by small foci of hemorrhage into the gray matter (agonal hemorrhage or handling artifact). Meningeal structures and nerve roots in these samples were absent, and some sections had slight fraying of the sectional margins, possibly a result of the removal of the meninges. Some sections had small tears most likely an artifact of excessive pressure during the ejection process. Overall architectural preservation in ejection samples was fair with quality score 1. (Fig. 2).

### Rats

In both groups of rat spinal cords examined, portions of meninges and peripheral structures were partially preserved, though to greater extent in samples collected via laminectomy. Architectural and cellular features in all sections from both groups were good to excellent (scores>1).

## Discussion

This study describes a standardized technique for the ejection of spinal cords from the spines of mice and rats as a valid alternative to the standard laminectomy procedure. While standard laminectomy yielded best results, sections obtained from samples collected by ejection were of similarly high quality, although not all morphological features were preserved to the same extent. For both collection methods, samples from rats had fewer histological artifacts than those of mice, presumably due to the larger size rendering them more robust and less vulnerable to mechanical damage. The most obvious benefit of the ejection method over laminectomy is the efficiency of the procedure. In general for both species, laminectomy can take on average between 25–35 minutes per animal (not including chilling time) depending on the skill level of the technician, while ejection takes no more than 5–6 minutes (less if perfusion is not performed). Not only is ejection significantly faster than laminectomy, it is also less sensitive to technical error. Conversely, laminectomy is preferable over ejection when structural integrity of the spinal cords’ margins, nerve roots, and meninges are the top priority, or when higher consistency is desirable among all samples collected.

Both laminectomy and ejection are advantageous over other commonly used methods of spinal cord isolation such as fixation and decalcification of the entire spinal column^1^ or dissection of the spinal cord from the vertebral column following 24 hours of fixation.^6^ Fixing and decalcifying spinal columns can yield concerns regarding overall turnaround time as well as the potentially undesirable effects of acid decalcification.^1^,^2^ If fresh samples are needed, this method cannot be utilized. Dissection of spinal cords from the bone following fixation is also much less efficient and though additional decalcification time is not necessary, fresh samples cannot be obtained.

For studies where large sample numbers and quick turnaround are essential, considerable time and effort may be conserved by the ejection method. Likewise, ejection may be beneficial for studies that require rapid preservation or utilization of tissue. On the other hand, laminectomy remains the method of choice for small studies without time constraints, and whenever peripheral structures (e.g. nerve roots) are to be preserved.
